# Elevated CSF levels of TACE activity and soluble TNF receptors in subjects with mild cognitive impairment and patients with Alzheimer's disease

**DOI:** 10.1186/1750-1326-6-69

**Published:** 2011-10-06

**Authors:** Hong Jiang, Harald Hampel, David Prvulovic, Anders Wallin, Kaj Blennow, Rena Li, Yong Shen

**Affiliations:** 1 Center for Advanced Therapeutic Strategies for Brain Disorders, Roskamp Institute, Sarasota, FL 34243, USA; 2 Department of Psychiatry, Psychosomatic Medicine & Psychotherapy, Goethe University, Frankfurt, Germany; 3 Department of Clinical Neuroscience, University of Göteborg, Sahlgren's University Hospital, Mölndal, Sweden; 4 Center for Hormone Advanced Science and Education Roskamp Institute, Sarasota, FL 34243, USA; 5 Haldeman Laboratory of Molecular and Cellular Neurobiology, Sun Health Research Institute, Sun City, AZ 85351, USA; 6 Current address: State Key disciplines: Physiology, Medical College of Qingdao University, Qingdao, China

**Keywords:** Alzheimer's disease, soluble tumor necrosis factor receptor, tumor necrosis factor converting enzyme, ADAM-17, biomarker

## Abstract

We recently reported that expression levels of tumor necrosis factor (TNF) receptors, TNFR1 and TNFR2, are significantly changed in the brains and cerebrospinal fluid (CSF) with Alzheimer's disease (AD). Moreover, we also found that, in an Alzheimer's mouse model, genetic deletion of TNF receptor (TNFR1) reduces amyloid plaques and amyloid beta peptides (Aβ) production through β-secretase (BACE1) regulation. TNF-α converting enzyme (TACE/ADAM-17) does not only cleave pro- TNF-α but also TNF receptors, however, whether the TACE activity was changed in the CSF was not clear. In this study, we examined TACE in the CSF in 32 AD patients and 27 age-matched healthy controls (HCs). Interestingly, we found that TACE activity was significantly elevated in the CSF from AD patients compared with HCs. Furthermore, we also assayed the CSF levels of TACE cleaved soluble forms of TNFR1 and TNFR2 in the same patients. We found that AD patients had higher levels of both TACE cleaved soluble TNFR1 (sTNFR1) and TNFR2 (sTNFR2) in the CSF compared to age- and gender-matched healthy controls. Levels of sTNFR1 correlated strongly with the levels of sTNFR2 (rs = 0.567-0.663, p < 0.01). The levels of both sTNFR1 and sTNFR2 significantly correlated with the TACE activity (rs = 0.491-0.557, p < 0.05). To examine if changes in TACE activity and in levels of cleaved soluble TNFRs are an early event in the course of AD, we measured these molecules in the CSF from 47 subjects with mild cognitive impairment (MCI), which is considered as a preclinical stage of AD. Unexpectedly, we found significantly higher levels of TACE activity and soluble TNFRs in the MCI group than that in AD patients. These results suggest that TACE activity and soluble TNF receptors may be potential diagnostic candidate biomarkers in AD and MCI.

## Introduction

Extensive evidence supports the concept that neuroinflammation plays a significant role in the neuropathogenesis of Alzheimer's disease (AD) [1-7]. Tumor necrosis factor-α (TNF-α) is one of the major inflammatory cytokines and plays a key role in AD [8] by regulating two TNF receptors, TNFR1 and TNFR2, through various signal transduction pathways, which finally converge to a common mechanism of neuronal death. We recently reported [9] that the expression levels of the two TNF receptors are significantly changed in AD brain tissues; specifically TNFR1 is increased whereas TNFR2 is significantly decreased in AD brains. Our studies on double transgenic mice demonstrate that deletion of TNFR1 in Alzheimer transgenic APP23 mice results in reduction of brain amyloid plaques and Aβ levels [10]. Furthermore, clinical evidence supports the concept that inhibiting the expression of TNF-α may result in cognitive improvement in AD patients [11,12].

TNF-α functions through binding to its two receptor subtypes, TNFR1 and TNFR2 [8]. Interestingly, studies have shown that both TNFR1 and TNFR2 can be shed from the cell surface with TACE/ADAM17 to become soluble forms [13]. TACE is a transmembrane disintegrin metalloprotease that cleaves precursor TNF-α to generate soluble, secreted TNF-α in macrophages and monocytes [14,15]. Both the cell-associated and the released forms of TNF are biologically active, but full inflammatory responses require the soluble form in at least some situations [16]. When TNFRs are shed by TACE, TNFR-mediated TNF-a signaling might be interrupted. Untill now, there are no reports regarding soluble TNFRs act as dominant-negative competitors to their receptor counterparts. Recently, soluble TNF receptors have been detected in CSF from preclinical AD patients [17]. However, it was not known whether TACE/ADAM-17 activity in the CSF would be changed. If TACE/ADAM17 is being shed from the brain cell membranes and leaks into the CSF after cleavage of the two TNF receptors, we would be able to detect TACE activity in the CSF throughout different stages of AD. Based on the evidence and rationales described above, we examined levels of TACE, and soluble TNF receptors in the CSF from AD patients and MCI subjects.

## Materials and methods

### Subjects

CSF samples were obtained from total 103 patients in two independent clinical research center: the Alzheimer Memorial Center, Department of Psychiatry, Ludwig-Maximilian University in Germany and the Department of Clinical Neuroscience, University of Goteborg, Sahlgren's University Hospital, Sweden. A total of 103 individuals were recruited from the above centers, 32 probable AD patients, 44 MCI subjects and 27 age-matched healthy individuals. As we described previously [18,19], diagnosis of AD was made according to the National Institute of Neurological and Communicative Disorders and Stroke (NINCDS-ADRDA) criteria [20], including the Mini-Mental State Examination (MMSE) and MCI was diagnosed according to the Petersen criteria [21]. MCI subjects performed 1.5 SD below the age-adjusted reference average in memory scales, as assessed using the Consortium to Establish a Registry for Alzheimer's Disease (CERAD) cognitive battery [22]. This battery included verbal learning, recognition, and recall tests; global cognitive function and activities of daily living were unimpaired. Controls were cognitively healthy individuals who underwent spinal anesthesia for surgery of the urinary tract or lower extremities. Psychiatric comorbidity was excluded by means of history, clinical examination, and Composite International Diagnostic Interview [23]. All the controls were cognitively normal according to Consortium to Establish a Registry for Alzheimer's Disease cognitive battery performance (within 1 SD in all subtests), and the participants had no complaints of cognitive impairment and had no history of dementia, did not show any signs of other psychiatric illnesses, and were followed up clinically for 3 years to exclude development of any neurodegenerative disease. To avoid spinal anesthesia as a potential confounding factor when collecting CSF, CSF was obtained immediately after inserting the needle and just before application of the anesthetic drug. CSF samples were obtained at baseline by lumbar puncture in the L3/L4 or L4/L5 interspace. The CSF samples were gently mixed to avoid possible gradient effects, centrifuged, and stored at -80°C pending biochemical analyses, without being thawn and re-frozen. All the procedures were approved by the institutional review boards (IRB) of the respective institutions, and consent forms were signed by the patients before sample collection.

### Assays of soluble TNFR (sTNFR) in the CSF

Both sTNFR1 and sTNFR2 were measured using a commercially available immunoassay according to the instructions provided by the manufacturer "R&D System" (Minneapolis, MN, USA). We added 50 μL of assay diluent to each well, then added 200 μL sample to each well, incubating for 2 hrs at room temperature. After aspirating and washing 3 times, 200 μL conjugate were added to each well and incubated for 2 hrs at room temperature. After aspirating and washing 3 times, we added 200 μL substrate solution to each well, incubated 20 min at RT, protecting from light. At last, we added 50 μL stop solution to each well, and read at 450 nm within 30 min. The detection limit of the assay is 7.8 pg/ml of human recombinant sTNFR with an inter-assay variation of < 10%.

### Assays of levels of TACE activity

TACE activity was measured using a solution-based assay. 7.5 μL of CSF was first diluted to 90 μL with running buffer (1 M Tris HCl, 10 mM ZnCl2). Ten microliter TACE substrate (R&D System, Minneapolis, MN, USA) was added to a final concentration. The enzymatic activity was measured using a microplate reader set to 320 nm of the excitation and 405 nm of the emission. The specific enzymatic activity of TACE is calculated FU/min.

### Statistical analysis

The Statistical Package for Social Sciences (SPSS 11.5 for Windows) was used for statistical comparisons. Results were presented as mean ± S.E.M. Paired-Samples T test was used to compare differences between means in two groups. Spearman's correlation coefficient was used for correlation analysis. A probability value of p < 0.05 was taken to indicate statistical significance.

## Results

### Levels of TACE activity and sTNFRs in CSF in the different diagnostic groups

As shown in Table 1, there was no statistically significant difference in age between the AD (71.4 ± 7.4) and the MCI group (71.9 ± 7.1) (p > 0.05). There was significant difference of age between the AD/MCI and the HC group (55.6 ± 10) (p < 0.05). There was no difference in gender distribution among the three groups (p > 0.01). MMSE scores differed significantly between AD patients (20.1 ± 4.8) and HC subjects (28.7 ± 1.1) p < 0.001), MCI patients (26.2 ± 2.2) and HC subjects (p < 0.05), and AD and MCI patients (p < 0.001).

**Table 1:** Baseline data in the patients with MCI, AD and the controls (Assay targets were in CSF)

Measured variables	Controls (n = 27)	MCI (n = 44)	AD (n = 32)
Age (years)	55.6 ± 10.0	71.9 ± 7.1^b^	71.4 ± 7.4^b^

Sex (M/F)	17/10	23/21	11/21

MMSE at baseline (0-30 p)	28.7 ± 1.1	26.2 ± 2.2^b^	20.1 ± 4.8^a^

CSF sTNFR1 (pg/mL)	705.0 ± 66.2	972.1 ± 50.7^b^	930.9 ± 52.5^b^

CSF sTNFR2 (pg/mL)	409.6 ± 130.2	602.6 ± 38.7^b^	832.1 ± 54.1 ^a^

CSF TACE activity (FU/min)	53.1 ± 6.6	91.2 ± 6.6^c^	82.0 ± 6.8 ^b^

Values are means ± S.D., except as noted otherwise. Abbreviations: AD patients with stable cognitive functions; MCI: mild cognitive impairment; Controls, healthy controls, with unimpaired cognition after at least 3 years follow-up; MMSE, mini-mental state examination; sTNFR, soluble tumor necrosis factor-α receptor; CSF, cerebrospinal fluid.

^a ^*p *< 0.001 vs. Controls.

^b ^*p *< 0.05 vs. Controls.

^c ^*p *< 0.01 vs. Controls.

**Figure 1:**
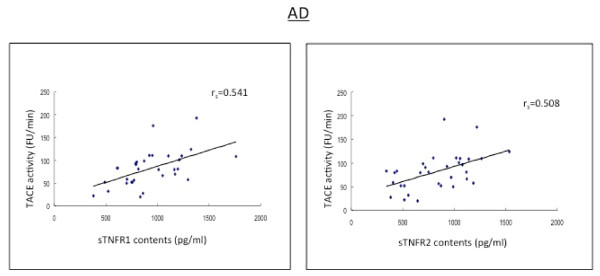
**The baseline levels of soluble TNF receptors, type I and type 2 (sTNFR1 and sTNFR2) in CSF correlate positively to the TACE activity in AD patients**.

Interestingly as shown in Figure 1, in MCI, TACE activity levels (91.2 ± 6.6 FU/min) and sTNFR1 (972.1 ± 50.7 pg/mL) did not fall in between AD (TACE: 82.0 ± 6.8 FU/min; sTNFR1: 930.9 ± 52.5 pg/mL) and HC subjects (TACE: 53.1 ± 6.6 FU/min; sTNFR1: 705 ± 66.2 pg/mL) (Table 1). There was a significant difference in TACE activity levels between AD and HC (df = 1; p < 0.001), between MCI and HC (df = 1; p < 0.01), and between AD and MCI patients (df = 1; p < 0.05). The sTNFR1 levels in the CSF from both AD and MCI patients were significantly higher than those in HC subjects (Paired-Samples T test, p < 0.05). There was a significant difference in sTNFR1 levels between AD and MCI (df = 1; p < 0.01), between AD and HC (df = 1; p < 0.05), and between MCI and HC (df = 1; p < 0.01). However, sTNFR2 levels in MCI patients (602.6 ± 38.7) fell into between AD patients (832.1 ± 54.1 pg/mL) and HC subjects (409.6 ± 130.2 pg/mL). Moreover, there was a significant difference in sTNFR2 of CSF between MCI patients and HC subjects (p < 0.05), and between AD and HC subjects (Paired-Samples T test p < 0.001).

### Significant correlations between baseline levels of sTNFR1 and sTNFR2 in CSF

**Figure 2:**
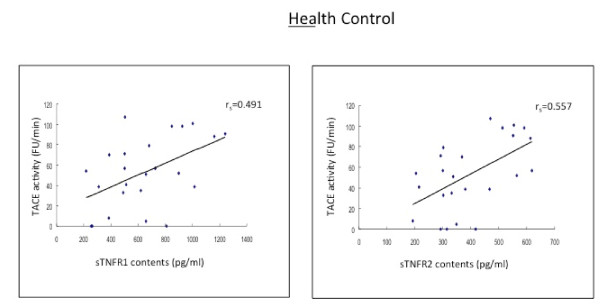
**The baseline levels of soluble TNF receptors type 1 and type 2 (sTNFR1 and sTNFR2) in CSF correlate positively to the TACE activity in aged-matched healthy individuals**.

**Figure 3:**
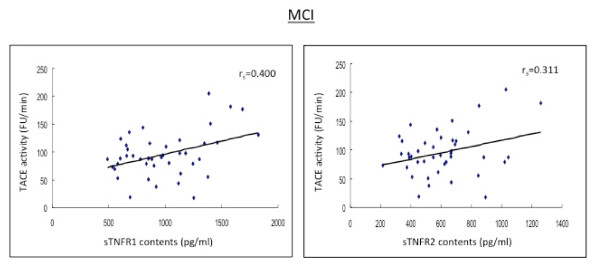
**The baseline levels of soluble TNF receptors, type 1 and type 2 (sTNFR1 and sTNFR2) in CSF correlate positively to the TACE activity in MCI patients**.

In the subjects with MCI, AD patients and HC subjects, there were positive and strong correlations between sTNFR1 and sTNFR2 in CSF (r_s _= 0.567-0.690, p < 0.01; r_s _= 0.567-0.663, p < 0.01) (Table 2 Figures 2, 3).

**Table 2:** Correlations between baseline CSF levels of soluble TNF receptors and TACE activity

CSF		TACE	sTNFR1	sTNFR2
Controls	sTNFR1	*r*_s _= 0.491^b^	/	*r*_s _= 0.567^c^
	
	sTNFR2	*r*_s _= 0.557^c^	*r*_s _= 0.567^c^	/

MCI	sTNFR1	*r*_s _= 0.400^c^	/	*r*_s _= 0.690^a^
	
	sTNFR2	*r*_s _= 0.311^b^	*r*_s _= 0.690^a^	/

AD	sTNFR1	*r*_s _= 0.541^c^	/	*r*_s _= 0.663^a^
	sTNFR2	*r*_s _= 0.508^c^	*r*_s _= 0.663^a^	/

All data were collected at baseline. Abbreviations: CSF, cerebrospinal fluid; sTNFR, soluble tumor necrosis factor-α receptor; AD: Alzheimer's disease; MCI: mild cognitive impairment; TACE: tumor necrosis factor-a converting enzyme.

^a ^*p *< 0.001.

^b ^*p *< 0.05.

^c ^*p *< 0.01.

### Correlations between the baseline levels of sTNFRs and TACE activity

**Figure 4:**
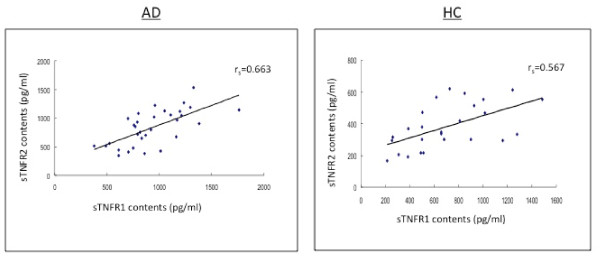
**The levels of soluble TNF receptor type 1 (sTNFR1) in CSF correlate positively to the soluble TNF receptor type 2 (sTNFR2) in the CSF between AD patients and aged-matched healthy controls**.

**Figure 5:**
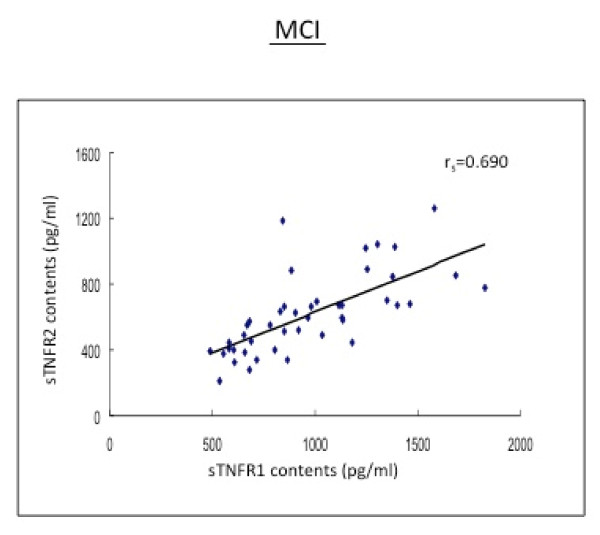
**The levels of soluble TNF receptor type 1 (sTNFR1) in CSF correlate positively to the soluble TNF receptor type 2 (sTNFR2) in the CSF among the MCI patients**.

The levels of both sTNFRs in CSF correlated strongly with the CSF activity of TACE (r_s _= 0.491-0.557, p < 0.05) (Table 2 Figures 1, 4, 5).

### Discussion

In the present study, as shown in Table 1, we did see the age difference between healthy controls and MCI and healthy controls and AD patients. Although up to now, based on the published literatures, there is no direct evidence for a possible impact of age on TACE, it is possible that aging may contribute to TACE changes via different mechanisms. In this study, we investigated TACE and both sTNFR1 and sTNFR2 in the CSF from AD and MCI patients. Interestingly, the levels of sTNFR in CSF were significantly increased in the subjects with AD compared to age and sex-matched healthy controls. Moreover, the CSF levels of both types of sTNFR in the AD subjects correlated strongly with TACE activity. These results suggest that the disease severity may affect the TACE activity.

Accumulating evidence supports the idea that neuroinflammation plays a significant role in the neuropathogenesis of AD [24]. Tumor necrosis factor-α (TNF-α) is one of the major inflammatory cytokines produced by activated astrocytes and microglia. Increasing evidence now supports the concept that excess TNF-α plays a central role in AD [8,25]. TNF-α exerts its biological effects by binding to TNFR1 and TNFR2. Both receptors belong to a super family of transmembrane receptors that are defined by a similar cysteine-rich extracellular domain, however, the intracellular regions of TNFR1 and TNFR2 appear to be unrelated, suggesting distinct modes of activation of signal transduction pathways [8,26,27]. TNFR1 is constitutively expressed at low levels on all nucleated cells, TNFR2 has a higher affinity for TNF than TNFR1 and binds TNF better at lower concentrations [9,28]. It has been proposed that in advanced stages of AD once extensive amyloid plaques have formed, elevated levels of pro-inflammatory cytokines, including TNF-α, may inhibit phagocytosis of toxic Aβ species and/or hinder efficient plaque removal by brain resident microglia [29]. Proinflammatory cytokines attenuate microglial phagocytosis stimulated by fAβ or complement receptor 3 and argue that this may, in part, underlie the accumulation of fAβ -containing plaques within the AD brain. The proinflammatory suppression of fAβ -elicited phagocytosis is dependent on nuclear factor kappaB activation [29]. In AD, excessive TNF-α activated TNFR1 through high affinity binding [9] and signaling accordingly suppresses Aβ degradation by reducing the expression of insulin degrading enzyme [30] and affects Aβ production through upregulation of BACE expression [31] and γ-secretase activity [32]. In our study, the significantly higher levels of sTNFR1 in CSF of the patients with AD compared to age-matched controls indicated that in spite of its conversion into soluble forms, the TNFR1 signaling pathway was still existing in the presence of excessive TNF-α, and may thus contribute to Aβ production as discussed above. On the other hand, the activation of the TNFR2 signaling pathway may be neuroprotective [33]. The elevated levels of sTNFR2 in CSF as shown in the present study suggest that TNFR2 is deficient as we recently reported [9], possibly cleaved by TACE. This impaired TNFR2 signaling pathway might alleviate the neuroprotective effects and also promote Aβ production.

We showed elevated levels of the TACE activity in CSF. Moreover, there were positive and significant correlations between both sTNFR levels and TACE activity in AD. TACE is a metalloprotease-disintegrin that releases several transmembrane proteins into soluble forms, including TNFR1 and TNFR2 [34,35]. Therefore, increased TACE activity would contribute to increased sTNFR as shown in the present study. These findings are in accordance with other reports that increased levels of TACE shed more sTNFRs [36,37].

Both but through different regulation mechanisms, β- (BACE) and α-(TACE) secretases contribute to Aβ production. BACE-mediated cleavage of amyloid precursor protein (APP) at the N terminus of the Aβ sequence is the first step in the generation of Aβ and BACE has been shown to be upregulated in sporadic AD brains [38,39]. TACE is also responsible for the α-secretase cleavage of APP [40,41]. Release of sAPPα precludes the formation of amyloidogenic peptides by BACE [42]. Although not presented in this study, there is general consensus that levels of CSF Aβ1-42 in AD are lower than those in ND [43]. The fact that TACE is increased in the CSF of AD might lead to the assumption that high TACE activity would parallel increased sAPPα and reduced Aβ levels. However, the most abundant form of Aβ - Aβ1-40 - is *not *decreased in AD, which speaks against a role of TACE activity in the modulation of CSF Aβ levels. The observed reduction of Aβ1-42 in CSF may be related to oligomerization and aggregation of Aβ1-42 rather than to TACE activities. Indeed, several independent studies demonstrate that modifying TACE activity does not affect APP cleavage by BACE and Aβ production [44-47]. This would explain why even increased TACE activity in AD it doesn't alter the Aβ production from APP. This was further supported by other reports that inhibition or knockdown of TACE does not affect sAPP formation and Aβ secretion under normal conditions [40,48]. TACE might not predominantly cleave APP in AD and plays a role only in the regulatory component of the α-secretase processing of APP. Therefore, we suggest that TACE may predominantly cleave TNFRs rather than APP, and contribute to upregulation of BACE and consecutive increase of Aβ production through the TNFR signaling pathway as discussed above.

In conclusion, this study shows elevated levels of sTNFRs and TACE activity in CSF, as well as correlations between them in patients with AD compared to age and sex-matched controls. These results further support the concept that the TNFR signaling pathway contributes to Aβ production and to the subsequent cytotoxicity in the brain. sTNFRs and TACE may become valid diagnostics targets to determine AD.

## Competing interests

The authors declare that they have no competing interests.

## Authors' contributions

HJ performed bioassays and wrote the manuscript draft. HH, AW and KB made clinical diagnosis for the subjects with MCI and patients with AD and also provided the CSF samples from these individuals. DP participated in manuscript drafting. HH and RL participated data analyses, experimental design discussing and manuscript drafting. YS initiated the project and designed the experiments and participated data analyses and overviewed manuscript drafting and finalized the paper. All authors have read and approved the final manuscript.
